# The Natural History Museum

**Published:** 1906-08-18

**Authors:** 


### The Hospital

A Journal of

The Medical Sciences and Hospital Administration.

Vol. XL.—No. 1,039. SATURDAY, AUGUST 18, 1906.

### THE NATURAL HISTORY MUSEUM.

The announcement publicly made by Dr. Ray Lankester, the Superintendent of the Natural History Department of the British Museum, that he has received notice of the intention of the trustees to call upon him to retire from his post next May, when he will attain the age of sixty, and that, if they are able to carry this intention into effect, he will receive a pension of no more than £300 a year, discloses a state of things which calls imperatively for reform. We learn from Dr. Lankester's letter that there are forty-five "trustees" of the British Museum, three of whom—the Archbishop of Canterbury, the Lord Chancellor, and the Speaker—are styled "principal" trustees, and that the whole body is accustomed to act through a standing committee of twenty. It appears, moreover, that the warrant appointing Dr. Lankester was signed by the three "principal" trustees, and entitles him to hold office until he is removed from it by these three trustees, or by two of them, by a notice given in writing. The resolution to call upon Dr. Lankester to retire was carried in the Standing Committee and afterwards confirmed at a meeting of the general body; but it does not appear what part, if any, the three principal trustees have taken in the matter. They certainly have not given Dr. Lankester the notice referred to, and, in the absence of this, the power of the general body to override the three seems to be doubtful, and to be one which may have to be settled in a court of law. It may well be the case that the circumstances of the appointment are such as to confer upon the holder an exemption from the ordinary operation of the Civil Service Superannuation Rules.

It was, no doubt, with some knowledge or prevision of what was intended, that Dr. Lankester, in his brilliant address as President of the British Association for the Advancement of Science, dwelt upon the unfortunate truth that the successive political administrators of the affairs of this country, as well as the permanent officials, have been unaware of the vital importance of the kind of knowledge which is called science, and of the urgent need for making use of it in public affairs. Dr. Lankester explained the position by the defective education, both at school and at university, of our governing class, as well as by a dislike among all classes to the support by public funds of posts which are beyond the reach of either popular clamour or of class privilege, and can only be held by men of special training and mental gifts. The existing position, indeed, is one of a revolt of ignorance against the dominion of knowledge, and, like servile revolts in general, it is in this instance characterised by an absolute disregard of the most elementary principles of honesty and fair play. In order to accept the position conferred upon him by the principal trustees, Dr. Lankester resigned the Linacre Professorship in the University of Oxford, tenable for life, with six months' vacation in the year, and a salary of £900; and all precedent was in favour of the supposition that he would be allowed to retain his Museum appointment for another ten years. It is superfluous to say that he is in full vigour of body and mind, or to say that his administration of the department over which he has presided has been marked by consummate ability and signal success. It is not difficult, reading between the lines of the transaction, to arrive at some conclusion with regard to its essential nature. The identity of forty-two of the trustees is "wrop in mystery." Their names are not given by "Whitaker," nor in any of the books of reference which we have searched for them. They are probably a series of ciphers, individually what is called "respectable," but deriving their sole value from the three "principal" figures at their head. They are presumably as unknown to science as they are to the public, and collectively willing, as a corporate body, to commit an injustice from which it is possible that each one of them individually would have recoiled. With regard even to the "principal" trustees, it should be remembered that gentlemen quite eligible to be Archbishops or Lord Chancellors are as plenty as blackberries, and that their eligibility does not imply any power of judging of the work of the eminent biologists who might perhaps be counted on the fingers of one hand. It is not difficult, for anyone who knows Dr. Lankester, to imagine that, from time to time, he may have shown insufficient respect for the dignity of ignorance; nor is it difficult to understand corporate incapacity to place the interests of the nation and of science above petty personal considerations. The importance of the question is not personal but national. Many of us are beginning to realise that the influence of ignorance and of obscurantism in the public councils is becoming a danger to the empire; and the political tendencies of the day are such that this danger is more likely to be increased than to be averted. We have already lost precious time by failing to equip ourselves with knowledge necessary for the competitions in which we are engaging or likely to engage; and our "governing" classes would send us into the field armed with bows and arrows against the artillery of modern science. The treatment of Dr. Lankester by the trustees of the Museum, and the subsequently disclosed treatment of Professor Judd by the Treasury, are but straws, but they show the direction of the current; and that direction is one against which, in the interests not only of national prosperity, but of national safety, it is the duty of all who possess knowledge to strive.

